# Resistance Training and Nutritional Supplementation in Older Adults with Sarcopenia after Acute Disease: A Feasibility Study

**DOI:** 10.3390/nu16183053

**Published:** 2024-09-10

**Authors:** Delky Meza-Valderrama, Dolores Sánchez-Rodríguez, Yulibeth Curbelo Peña, Cindry Ramírez-Fuentes, Elena Muñoz-Redondo, Andrea Morgado-Pérez, Norma Ortíz-Agurto, Paola Finis-Gallardo, Ester Marco

**Affiliations:** 1 Rehabilitation Research Group, Hospital del Mar Medical Research Institute, 08024 Barcelona, Catalonia, Spain; doloresmaria.sanchez-rodriguez@chu-brugmann.be (D.S.-R.); emarco@psmar.cat (E.M.); 2 Physical Medicine and Rehabilitation Department, National Institute of Physical Medicine and Rehabilitation, Panama City 0819, Panama; 3 Physical Medicine and Rehabilitation Center, Ciudad de la Salud, Caja de Seguro Social, Panama City 0819, Panama; 4 Sistema Nacional de Investigación (SENACYT), Panama City 0824, Panama; 5 Geriatrics Department, Brugmann University Hospital, Université Libre de Bruxelles, 1050 Brussels, Belgium; 6 Physical Medicine and Rehabilitation Department, Parc de Salut Mar (Hospital del Mar, Hospital de l’Esperança), 08024 Barcelona, Catalonia, Spain; 7 Faculty of Health and Life Sciences, Metropolitan University of Education, Science and Technology (UMECIT), Panama City 0819, Panama; 8 Faculty of Health and Life Sciences, Universitat Pompeu Fabra, Dr. Aiguader Building (Mar Campus), Dr. Aiguader 80, 08003 Barcelona, Catalonia, Spain

**Keywords:** sarcopenia in older adults, feasibility, resistance exercise, hydroxy-methyl-butyrate, protein supplementation

## Abstract

Resistance exercise and protein supplementation are recognized as effective treatment strategies for age-related sarcopenia; however, there are limited data on their feasibility, tolerability, and safety. The primary outcome of this study was feasibility, evaluated through the 15-item TELOS (Technological, Economics, Legal, Operational, and Scheduling) feasibility components and by recruitment, retention, and consent rates. Tolerability was measured by examining permanent treatment discontinuation, treatment interruption, exercise dose modification, early termination, rescheduling of missed sessions, losses to follow-up, attendance, and nutritional compliance. Safety was evaluated using the parameters provided by the European Medicines Agency, adapted for exercise interventions. Thirty-two subjects were recruited (average age 81.6 [SD 9.3] years). The TELOS components were assessed before the intervention; out of 15 questions relevant for successful implementation, 4 operational needs answers required specific actions to prevent potential barriers. The recruitment rate was 74%. Eleven patients (34.4%) had permanent treatment interruption (retention rate = 65.6%). Patients attended a mean of 23 (SD 12.0) exercise sessions, with a mean of 56 (SD 32.6) nutritional compliances. A total of 21 patients (65.6%) experienced adverse events unrelated to the intervention, while 7 patients (21.9%) presented adverse reactions to strength exercise. The main barriers to feasibility were operational components and recruitment challenges. Although the intervention was generally safe, the high rate of probable adverse effects, unrelated to the intervention but associated with the individual’s baseline health condition, may affect adherence to treatment programs of this kind.

## 1. Introduction

Sarcopenia, the loss of muscle mass and strength in adults, is now recognized as a complex phenomenon of multifactorial etiology associated with adverse outcomes, disability, and mortality in older adults [1]. The Task Force of the International Conference on Frailty and Sarcopenia Research (ICFSR) reaffirms that exercise and nutritional interventions are the primary treatment for older adults with sarcopenia [2]. Although diverse substances are being studied for their potential positive effects on muscle health, the most significant muscle mass and strength improvements in individuals with sarcopenia have been achieved with moderate-to-high-intensity resistance training. Beyond physical activity [3,4,5,6,7,8], current guidelines strongly recommend progressive resistance training for the prevention and treatment of sarcopenia [7,9]. The evidence supporting protein supplementation such as β-hydroxy-β-methyl-butyrate (HMB) remains insufficient to validate a general recommendation for its use [9]; however, supplementation with 3 g of HMB has been particularly beneficial in improving muscle strength and body composition in individuals aged 65 and older, as a key regulator of muscle protein anabolism [10,11,12,13].

The patients’ rehabilitation process starts with clinical and functional assessment; then, objectives and a therapeutic plan to achieve them are established. Available resources in each physical environment, functional status, and patient comorbidities can modify exercise intensity and tolerance [14], particularly in older patients [15]. Therefore, although four types of exercises are recommended for older adults—strength, endurance, balance, and flexibility [16]—progressive resistance exercise has been suggested as the first-line treatment for these patients [9]. The characteristics of exercise-based rehabilitation programs are not often described in the medical literature [17]; there is a need to standardize the wide range of goals and therapies [18,19] to provide the external validity necessary for clinical trials [17]. Thus, the complex variety of goals and therapies has been called the “black box” of rehabilitation [19]. As well as testing the efficacy of an intervention through a clinical trial, we must verify that it can be carried out as proposed in clinical settings [20].

Implementing exercise interventions is a challenge in older populations because their medical and social considerations may limit tolerability and adherence. There is a need for an adequate methodology to standardize the description of rehabilitation interventions [18] and provide quality evidence to understand challenges, limitations, barriers, and facilitators before assessing the effectiveness of a specific intervention [21]. Feasibility studies fit this need well. They are defined as a process used to determine if a suggested method, plan, or piece of work is possible or reasonable [22]. These studies are crucial to answering the question “Can it work?” They help researchers understand the barriers and facilitators that may impact the success of their research [23]. Feasibility studies also aid in decision making regarding the implementation of interventions [24]. Therefore, they may facilitate the development of more effective rehabilitation interventions [23], particularly complex interventions and multidisciplinary programs [25].

The “Postacute Sarcopenia: Supplementation with HMB After Resistance Training” (PSSMAR) study is a randomized, double-blind, placebo-controlled clinical trial designed to evaluate the effectiveness of adding nutritional supplementation to an exercise-based intervention in post-acute patients with sarcopenia. Previous data from this study indicated that supplementation with three g/day of HMB alongside a progressive resistance exercise program significantly improved muscle strength and physical performance in older women with sarcopenia during the post-acute phase following hospitalization [26]. However, the study encountered several limitations, particularly a high attrition rate. This was likely influenced by factors such as the advanced age of participants, the prevalence of comorbidities, and the challenges associated with recruiting and following up with patients recently discharged from the hospital. These limitations underscore the inherent difficulties in conducting sarcopenia trials within this population and highlight the need to address key issues such as enrollment, participation, and retention of older patients in resistance exercise programs [27]. More high-quality data on the feasibility, tolerability, and safety of evidence-based interventions for sarcopenia is needed. Addressing this knowledge gap is critical for identifying key barriers and facilitators in clinical trials, as well as for assessing the effectiveness of sarcopenia interventions and their implementation in clinical practice. Based on these considerations, this study aimed to build on these findings by specifically assessing the feasibility, tolerability, and safety of a 12-week rehabilitation program, which included muscle resistance exercises and HMB supplementation for older adults with sarcopenia during the hospitalization period following an acute illness.

## 2. Materials and Methods

### 2.1. Study Design

This is a feasibility study with data from the PSSMAR clinical trial in older adults with sarcopenia after discharge from a post-acute geriatric rehabilitation unit. The PSSMAR recruitment period spanned from January 2017 to January 2021, with an interruption due to the COVID-19 pandemic from March 2020 to September 2020 (clinicaltrials.gov registration: NCT02679742) [26]. 

### 2.2. Participants

Eligibility criteria for participants in the PSSMAR study have been comprehensively detailed in both the study protocol [28] and the original research publication [26]. These criteria ensured a rigorous selection process to identify suitable candidates for the intervention. Selection criteria for the present study were as follows:

#### 2.2.1. Inclusion Criteria

Men and women aged 60 years and older with sarcopenia diagnosis who had been discharged from a post-acute geriatric rehabilitation unit within the previous three months were eligible for inclusion. Sarcopenia diagnosis followed EWGSOP2 criteria: low muscle strength (grip strength < 16 kg for women, <27 kg for men, or Chair Stand test > 15 s for five rises) and low muscle mass (<80% of reference data for the European population). Cognitive ability to understand study procedures was required (proven by a Mini-Mental State Examination score ≥ 21) [1,29,30].

#### 2.2.2. Exclusion Criteria

Participants with active malignancy (except basal or squamous cell skin carcinoma and carcinoma in situ of the uterine cervix), life-compromising clinical conditions, major lower limb surgery within the past six months, participation in other exercise programs within the past six months, contraindications to resistance exercises, use of medications interfering with the nutritional intervention (e.g., steroids, free amino acid supplements), and allergies, intolerances, or contraindications to Ca-HMB [31,32].

### 2.3. Settings

This study was performed in the Physical Medicine and Rehabilitation Department of a university hospital in Barcelona (Catalonia, Spain). 

### 2.4. Intervention

**Figure 1:**
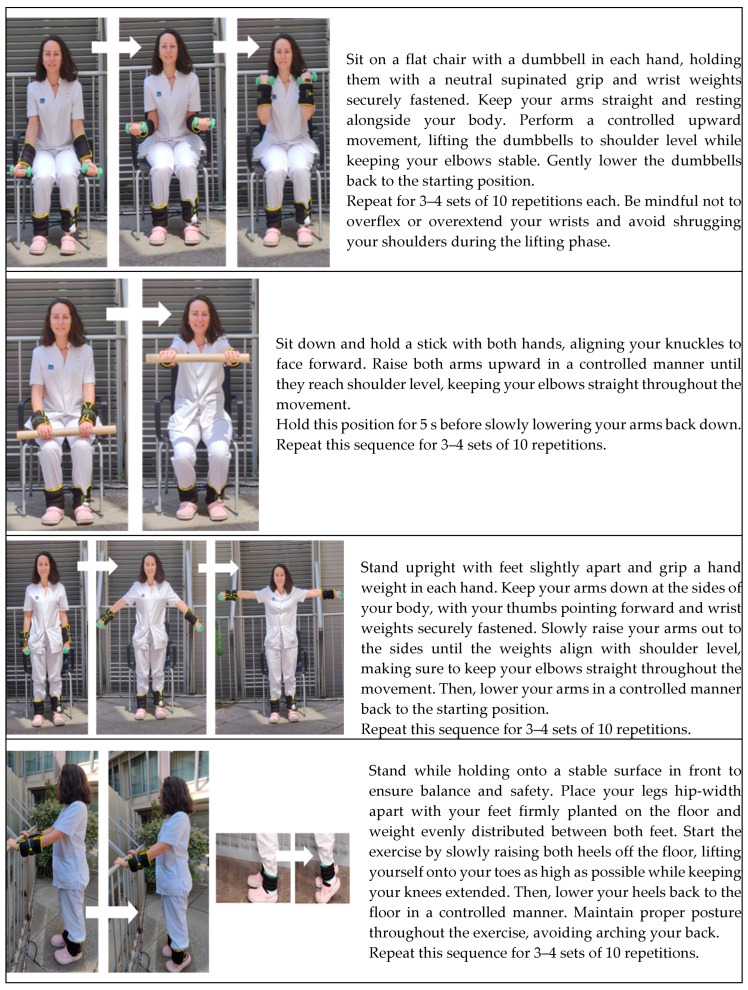

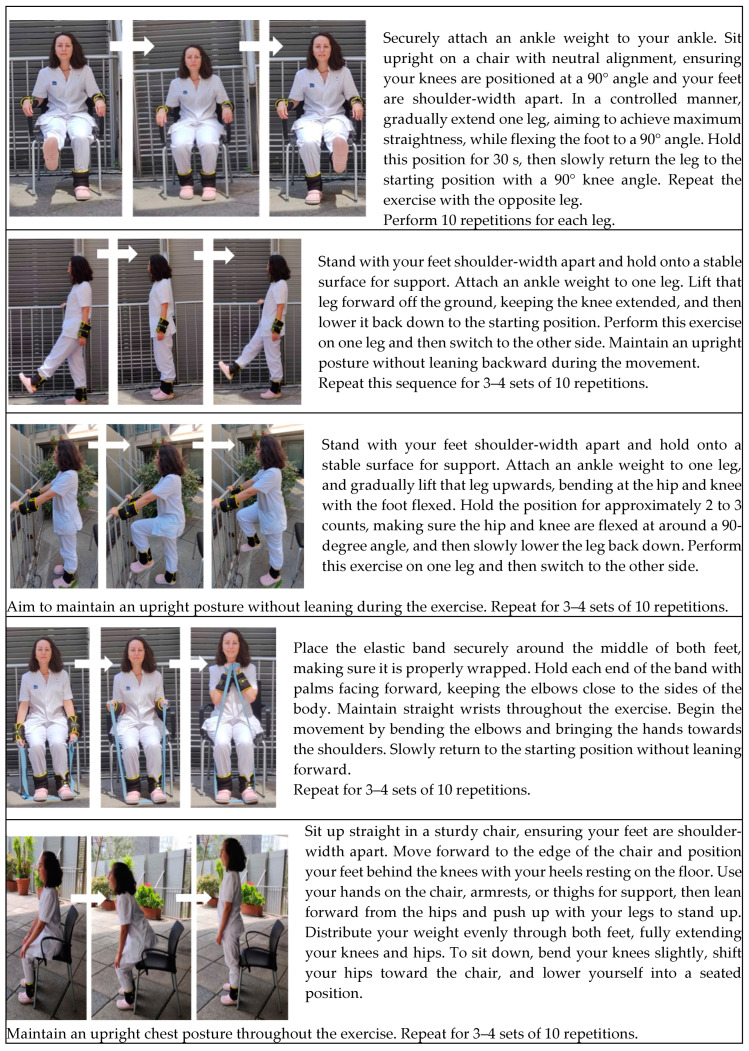
The PSSMAR exercise protocol. Note: Comprehensive progressive strength exercise program for participants, adapted from Meza-Valderrama et al. Arch Gerontol Geriatr. 2024 Apr:119:105323. doi: 10.1016/j.archger.2023.105323 [26], with permission from Elsevier.

**Figure 2:**
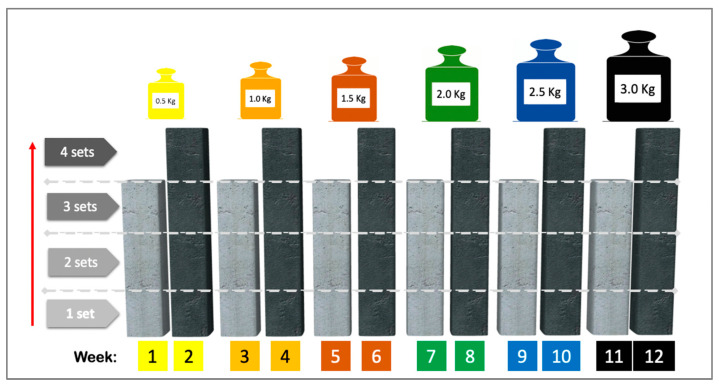
Exercise protocol intervention: diagram showing the progression of loads and exercise volumes. Note: Training started with a load of 0.5 kg on each limb and increased to 0.5 kg every two weeks until 3.0 kg was reached at the end of the intervention (if tolerated). Each new load began with three sets of 10 repetitions in the first week and four sets of 10 repetitions in the second week. Reproduced from Meza-Valderrama et al. Arch Gerontol Geriatr. 2024 Apr:119:105323. doi: 10.1016/j.archger.2023.105323 [26], with permission from Elsevier.

The Template for Intervention Description and Replication (TIDieR) checklist describes the exercise-based intervention [33]. The training protocol (Figure 1) consisted of a combination of muscle resistance and balance exercises supervised by a physical therapist (36 one-hour sessions, i.e., 3 sessions per week for 12 weeks). Muscle resistance exercises of the upper and lower limbs (shoulder flexor and abductor, elbow flexor, hip flexor and abductor, knee flexor and extensor and plantiflexor muscles) were performed according to the principles of periodization and progression [6]. Training started with 0.5 kg on each limb and increased every two weeks by 0.5 kg, according to the participant’s tolerance (see Figure 2). Each session began with a 5 min warm-up followed by 30 min of resistance training (10 min, upper limbs; 20 min, lower limbs), 10 min of balance exercises, and a 5 min cool-down. After each exercise session, patients received 3 g/day of HMB for 12 weeks (84 packets, a total dose of 252 g) and were instructed to consume a daily dose at the same time on non-training days during the 12 weeks.

### 2.5. Primary Outcome

The primary outcome was feasibility, which was assessed via the technological, economic, legal, operational, and scheduling (TELOS) methodology, a comprehensive tool designed for feasibility assessment in biomedical research studies [34]. The TELOS components were adapted from previous studies [20,35]. The research team agreed on which items should be considered for each TELOS component before study commencement; 15 questions and their expected answers were agreed upon. The procedure was considered feasible if all the answers were those expected; in case of discordance, strategies to address eventual barriers were carried out [35,36,37]. Other parameters related to the study considered indicative of feasibility were recruitment, retention, and consent rates. These were calculated using the following formulas [38]:


$$\mathrm{Recruitment}\;\mathrm{rate}=\frac{\mathrm{Number}\;\mathrm{of}\;\mathrm{recruited}\;\mathrm{participants}}{\mathrm{Recruitment}\;\mathrm{time}\;(\mathrm{months})}$$

$$\mathrm{Retention}\;\mathrm{rate}=\frac{\mathrm{Number}\mathrm{of}\;\mathrm{participants}\;\mathrm{included}\;\mathrm{in}\;\mathrm{the}\;\mathrm{analysis}}{\mathrm{Number}\mathrm{of}\;\mathrm{participants}\;\mathrm{recruited}\;\mathrm{and}\;\mathrm{randomized}}\times 100$$

$$\mathrm{Consent}\;\mathrm{rate}=\frac{\mathrm{Number}\mathrm{of}\;\mathrm{participants}\;\mathrm{included}\;\mathrm{in}\;\mathrm{the}\;\mathrm{analysis}}{\mathrm{Number}\mathrm{of}\;\mathrm{elegible}\;\mathrm{participants}}\times 100$$

### 2.6. Secondary Outcomes

Tolerability and safety were considered secondary outcomes. For tolerability, all participants were expected to complete at least 25 (70%) exercise sessions and consume at least 59 (70%) sachets of nutritional supplementation. Tolerability was assessed using variables adapted from oncology drug trials [39]: (a) permanent discontinuation of resistance exercise and/or protein supplementation before completing at least 70% of the total study intervention protocol; (b) treatment interruption: patients missing at least three consecutive sessions or at least three intakes of supplementation, but who completed more than 70% of the total study intervention protocol; (c) dose modification: patients requiring exercise dose reduction during training; (d) early termination: patients who finished the program early but completed more than 70% of the total study intervention protocol; and (e) re-scheduling of missed sessions, which was permitted within the study intervention period. Conventional intervention-related tolerability variables were as follows: losses to follow-up (the number of patients who did not complete the follow-up assessments), attendance (number of attended sessions), and nutritional compliance (number of sachets consumed during the 12-week intervention).

Safety was studied using the parameters provided by the European Medicines Agency [40] adapted for exercise interventions: (a) adverse events: untoward medical occurrences in patients that do not necessarily have a causal relationship with the study intervention; (b) adverse reactions: noxious and unintended responses to exercise or supplementation which occurs at intensities normally used in sarcopenia therapy; (c) unexpected adverse reactions: adverse reactions of a severity that is not consistent with study intervention; and (d) serious adverse events: any untoward medical occurrences that result in death, are life-threatening, require hospitalization or prolongation of existing hospitalization, or result in persistent or significant disability. Safety was evaluated by the frequency of these events/reactions occurring during any supervised session or throughout the trial.

### 2.7. Other Variables

The following demographic, functional, and clinical variables were also collected: age, sex, body mass index, handgrip strength, fat-free and fat mass indexes; physical performance was assessed by the gait speed test (measured in m/s) and the Short Physical Performance Battery (SPPB, measured in points), prevalence of malnutrition according to the Global Leadership Initiative on Malnutrition (GLIM) criteria [41], comorbidity by the Charlson index [42,43], functional status using the Barthel index [44] and Lawton index [45], and quality of life with the Sarcopenia and Quality of Life (SarQoL) questionnaire [46]. 

The SPPB is an assessment tool consisting of three tests: tandem standing balance, 4 m gait test, and chair stand test. Each test scores from 0 to 4 points. The total SPPB score ranges from 0 to 12 points, with higher scores indicating better performance. A small meaningful change was defined as an improvement of 0.5 points and a substantial change as an improvement of 1 point at 12-week follow-up [9,47,48].

### 2.8. Statistical Analysis

Data were reported as means and standard deviations (SDs) for quantitative variables and absolute and percentage values for categorical variables. The assumption of normality was estimated with the Shapiro–Wilk test. Univariate analysis was performed using Chi-square or Fisher tests for categorical variables and Student’s *t*-test for independent samples (intergroup analysis) and related samples (intragroup analysis); mean differences were reported with their 95% confidence intervals (95%CI). The significance level was set at *p* ≤ 0.05. Data were analyzed using IBM SPSS Statistics v.28 (SPSS Inc., Chicago, IL, USA).

## 3. Results

**Figure 3:**
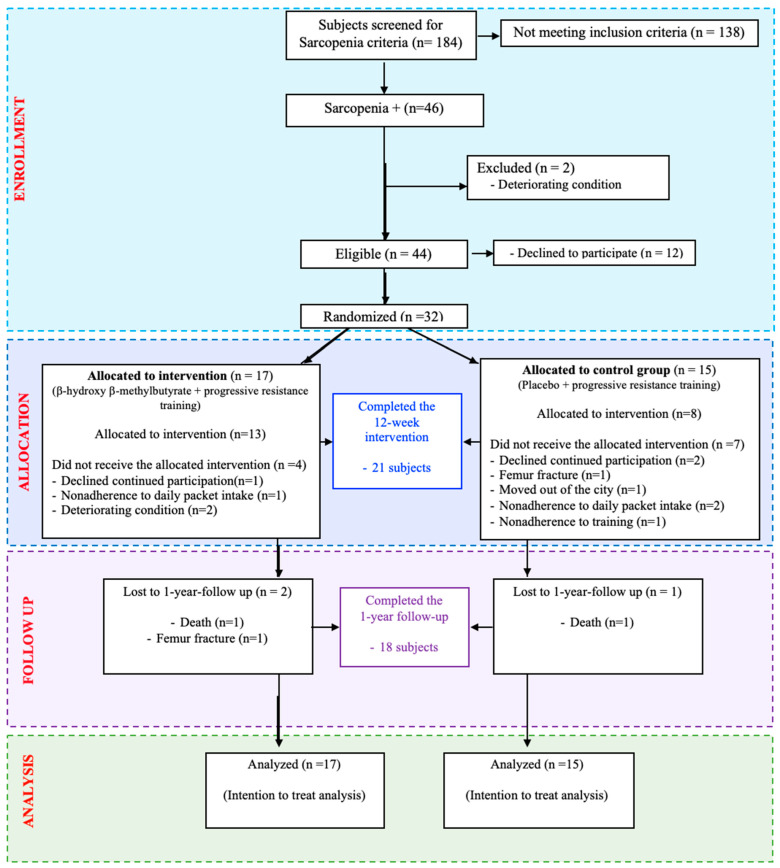
Recruitment flowchart of the PSSMAR study (n = 32).

Thirty-two participants were included in the PSSMAR study (aged 81.6 [SD 9.3] years; 75% women). A recruitment flowchart is shown in Figure 3. The demographic, functional, and clinical characteristics of participants are summarized in Table 1.

Table 2 shows the main results of a previously published study, which showed a significant enhancement in muscle strength and physical performance among older women dealing with sarcopenia after supplementation of 3 g/day of HMB with a progressive resistance exercise program. The handgrip strength, chair-stand test, and Total SPPB test in the intervention group showed an improvement in the intragroup analysis. The intergroup analysis revealed a significant improvement in the intervention group’s balance test and total SPPB. The study observed “substantial changes” and an improvement of 1 point in the SPPB total scores after a 12-week follow-up using the thresholds of meaningful change in physical performance proposed by other authors [9,47,48]. Considering the limited number of men, the authors conducted an intention-to-treat analysis focusing on the subsample of women. The rest of the variables studied showed no significant changes [26].

The TELOS components were evaluated before the initiation of baseline assessments. Of the 15 questions considered relevant to assess feasibility, 7 answers were “unknown” or not as expected; these were specifically addressed by describing actions to overcome potential barriers to implementation, as shown in Table 3. No significant differences were found between groups when assessing the feasibility variants. The recruitment rate was 0.74 participants per month (32 participants/43 months), the retention rate was 65.6%, and the consent rate was 47.7%. 

The variables for assessing tolerability and safety are shown in Table 4. The reasons for permanent treatment discontinuation, treatment interruption, exercise dose modification, early termination, and re-scheduling of missed sessions are detailed as follows:

(a) The reasons for permanent treatment discontinuation were deterioration in general condition (two participants in each group), supplement had an unpleasant taste (two participants in each group), lack of interest in continuing (one participant in the control group), moving out of the city (one participant in the control group), fear of being infected by Coronavirus disease (COVID-19) (one participant in the control group); and lack of family support for attending the program (the second reason for one participant in the control group).
(b) Treatment interruption: Among the participants who completed 70% or more of the intervention, six participants (18.8%, four participants in the intervention group and two participants in the control group) interrupted the exercise program, while four (12.5%, two participants in each group) interrupted the supplementation. The causes were exacerbated chronic low back pain, COVID-19 onset, pulmonary disease exacerbation, and holidays. No significant differences were found between the groups.
(c) Exercise dose modification: Six participants (18.8%, three participants in each group) required modification of some parameter of the progressive resistance exercise program; the reasons for this are listed in Table 5.
(d) Early termination: Nine participants (28.1%) ended participation earlier than planned for the following reasons: sudden onset of COVID-19 (two patients in the intervention group and one in the control group), exacerbation of chronic disease (one patient in the control group), and reasons unrelated to the intervention, such as holidays and vacations (four in the intervention group and one in the control group).
(e) Re-scheduling of missed sessions: Six participants rescheduled 21 sessions (18.8%, three patients in each group).

The reasons for being lost at 1-year follow-up were deterioration of general condition (one patient in the intervention group), femoral fracture (one patient in the intervention group), death (one patient in each group), non-attendance for follow-up (one patient in the intervention group), and fear of going to the hospital due to the risk of COVID-19 infection (one patient in each group). Patients attended a mean of 23 (SD 12.0) sessions; nutritional compliance, as represented by average intake of 56 sachets (SD 32.6), fell short of the expected minimum of 59 sachets. No significant differences were found between groups when assessing the variants of tolerability.

Concerning the safety of the intervention, among the adverse events detected (not related to the intervention) were falls, femur fracture or sprained ankle in a fall, pneumonia requiring hospitalization, deterioration of the general condition, cognitive deterioration, exacerbation of the chronic disease, cancer diagnosis, cataract surgery, emotional decline due to death of a close relative, hospitalization for COVID-19, and death. Adverse reactions related to exercise were localized muscle pain, fatigue, cervical pain, exacerbated low back pain, and joint pain in shoulders and knees. These adverse reactions were low-intensity; none of the patients had to stop the exercise intervention. No adverse reactions of supplementation, unexpected adverse reactions, or serious adverse reactions were observed during the study period. No significant differences were found between groups when assessing the variants of safety.

## 4. Discussion

This study evaluates the feasibility, tolerability, and safety of resistance training combined with nutritional supplementation in older adults with sarcopenia during the post-acute period following recent hospitalization. It opens up ‘’the black box in rehabilitation’’ by providing a detailed description of the processes during the study period. The study covers a significant research gap and identifies barriers and facilitators to implementing evidence-based interventions in sarcopenia. This information may be useful for developing clinical trials and implementing interventions in clinical practice. 

The authors must point out that other treatments besides physical exercise and protein supplementation are being explored. Diverse substances (e.g., testosterone, estrogens in women, thyroid hormones, 25(OH)D3, insulin, leptin, myostatin inhibitors, and others) are currently being studied for their potential positive effects on muscle health [49]. Despite ongoing research, regulatory agencies such as the U.S. Food and Drug Administration and the European Medicines Agency have not yet approved any drugs for the treatment of sarcopenia. Thus, physical exercise and nutritional interventions remain the available treatment for patients with sarcopenia [9,50]. 

In this context, it is important to note the effectiveness of adding nutritional supplementation to an exercise-based intervention, resulting in significant SPPB improvement at 12-week follow-up. Moreover, women who received HMB supplementation performed significantly better in the follow-up chair stand SPPB and total SPPB tests [26]. The present study also demonstrated “substantial changes” in total SPPB scores after a 12-week follow-up, according to established thresholds of meaningful change in physical performance [26,47]. 

Regarding muscle strength, intragroup analysis revealed a significant increase in handgrip strength among women in both the intervention and control groups, with gains of 3.7 kg and 1 kg, respectively [26,47]. In a recently published study [26], we revised our sample size estimation to check the impact of attrition on the effectiveness of this intervention. With respect to physical performance and muscle strength, our revised analysis supports the effectiveness of our approach in assessing the feasibility, tolerability, and safety of this post-hospitalization nutritional and exercise program intervention in older adults with sarcopenia [51]. 

Assessing the TELOS components highlights the critical role of economic and social support for older adults in such interventions. In our study, significant challenges were identified in this respect, mainly within the operational and scheduling components. Specifically, responses to four out of six questions related to operational needs and two out of three questions within the scheduling section were marked as “unknown or unexpected”. The reported operational barriers encompassed issues such as patient enrollment, acceptance of the intervention by patients and their support network and social limitations that hindered the participation of older adults. To address these challenges, we proposed measures such as the involvement of relatives in the rehabilitation process and the implementation of educational strategies to highlight the importance and benefits of participating in research studies. This emphasis on education aims to empower healthcare professionals, patients, and their families, thereby raising awareness about the advantages of exercise. These strategies aligned with findings from an umbrella review that identified key factors associated with adherence to physical exercise in older adults with chronic diseases. The review highlighted the significance of participant education, realistic expectations, knowledge about risks and benefits, multidisciplinary professional involvement, and social support as critical factors for enhancing adherence to physical exercise [52]. Other previous studies have cited similar barriers, such as environmental factors and resources [15], lack of nearby facilities [53], absence of companionship [54], concern about physical health, lack of motivation, fear of falls [55], negative attitudes among patients and caregivers [56], as well as the low priority given by physicians to nutrition and the uncertainties about their professional roles in delivering nutritional advice and care [57].

Among the actions taken to address potential scheduling barriers, we determined the need to provide evidence to patients, their relatives and health professionals about the realism and potential benefits of the intervention in a post-hospitalization period; therefore, we proposed informing patients about the necessity of timely treatment for sarcopenia, specifically resistance exercise combined with nutritional intervention. Along this line, previous publications highlighted frequently reported barriers, such as the low clinical priority given to malnutrition during patient hospitalization [58] and the limited time available for healthcare providers to discuss dietary issues with patients [59]. These barriers were compounded by a lack of awareness and understanding of resistance exercise among older adults [60]. To mitigate the barriers, our study emphasizes the importance of designing similar interventions with attention to preservation of the older adult’s social and physical autonomy and the enhancement of education regarding the benefits and importance of rehabilitation programs. This approach can facilitate decision making about participation in future clinical trials and exercise programs in usual care. 

Our study also evaluates feasibility by examining recruitment, retention, and consent rates. In the context of older patients with sarcopenia following acute illness, the recruitment rate of 0.74 participants per month is acceptable, even though it is lower than those reported in other clinical trials performed in community settings (median = 2.44, IQR: 0.62–6.41) and placebo-controlled trials (median = 0.84, IQR: 0.38–1.93). However, the retention rate of 65.5% and consent rate of 47.7% are notably lower than the medians of 88% (IQR: 80–97%) and 72% (IQR: 50–88%), respectively, in those settings [38].

Regarding tolerability, it is noteworthy that nearly 34% of the participants in our study experienced permanent treatment discontinuation, a percentage significantly higher than the 12.7% reported in the “Implementing an Exercise Intervention in Ambulatory Older Adults” study [61]. It is important to note that the comparability of the two studies is limited due to differences in participant characteristics. We have not found similar studies conducted in populations of older adults in the subacute period of an illness. In our study, permanent treatment discontinuation was due to external factors affecting the patient, which highlights the need to count on family and/or environment support for a successful clinical trial.

The safety analysis revealed that 21.9% of participants experienced minor anticipated adverse reactions to resistance exercise, with no treatment interruptions. However, exercise dose adjustments were necessary for 18.8% of patients, consistent with previous research [7,62], indicating the safety of progressive resistance exercise in older adults. In our study, a significant proportion (65.6%) of reported adverse events were unrelated to the intervention. These included falls, hospitalizations due to chronic diseases, or deterioration of general condition. This finding aligns with the existing literature, which cites poor health as a frequent barrier to physical activity [54]. These events can be attributed to post-discharge sarcopenia complications, which are associated with worsened physical function and reduced home discharge rates [51]. Non-participation reasons mainly stemmed from logistical challenges in accessing the intervention setting, highlighting the necessity of educational initiatives to inform patients and their families about disease significance, treatment implications, and commitments related to research participation.

Several strengths should be highlighted. First, this study addresses a crucial gap by addressing the feasibility of sarcopenia interventions in older individuals during the post-acute period. Our findings are expected to inform future trials and guide the implementation of rehabilitation programs in clinical practice. The study also stands out for its high-quality methodology, featuring an appropriate study design incorporating various feasibility, tolerability, and safety criteria, evaluated according to updated European Medicines Agency criteria tailored to physical interventions. Given the scarcity of feasibility studies in this field, the innovative and transparent methodology employed here is offered as a reference point. Moreover, these results highlight several critical insights for future studies and clinical guidelines. The substantial rate of adverse events unrelated to the intervention and the high rate of permanent treatment discontinuation due to external factors suggest that interventions should be initiated much earlier. 

Shifting the focus from post-hospitalization to pre-hospitalization interventions can make it possible to address deterioration due to chronic diseases before they become more advanced [63,64,65]. This proactive approach would likely increase the decision-making autonomy of individual patients regarding their functional recovery measures, including exercise and nutrition, rather than depending heavily on familial, economic, and social support. Therefore, researchers should be aware of the need for opportune intervention. Implementing programs during an earlier stage of disease progression could improve adherence rates and overall outcomes, making interventions more effective and sustainable.

Three limitations should be acknowledged. The study’s specific target population and limited sample size may restrict the generalizability of its findings. The tolerability and safety assessments were tailored specifically to this intervention and may not apply to different interventions. Finally, the TELOS feasibility model involves some subjectivity in assessment and interpretation, potentially affecting the reproducibility of findings. Nonetheless, the authors consider this a minor limitation, given the solid evidence supporting the rational approach of TELOS in feasibility studies, which has demonstrated its utility in clinical nutrition and rehabilitation trials [20,36,37].

## 5. Conclusions

Progressive resistance exercise combined with protein supplementation is a feasible and tolerable intervention in older adults with sarcopenia detected after hospitalization in post-acute care. However, significant operational challenges remain the primary barriers to feasibility. Retention and consent rates are the biggest challenges in this study population. While the intervention is generally safe, a high rate of adverse effects—unrelated to the intervention but linked to the participants’ baseline health conditions—may decrease adherence to these treatment programs. Future studies and larger sample sizes are needed to address operational and health-related barriers if we are to enhance participation and retention, successfully implement similar programs, and maximize their efficacy and adherence.

## Figures and Tables

**Table 1:** Baseline demographic and clinical characteristics of the study participants.

	Total Sample (n = 32)
Age (years), (mean ± SD)	81.6 ± 9.3
Sex, women (%)	24 (75%)
Body mass index (kg/m^2^), (mean ± SD)	24.2 ± 4.5 (women); 21.6 ± 3.3 (men)
Handgrip strength (kg), (mean ± SD)	12.9 ± 4.2 (women); 22.7 (± 4.1) (men)
Fat-free mass index (kg/m^2^), (mean ± SD)	12.9 ± 1.8 (women); 14.0 ± 2.0 (men)
Physical performance:	
Short Physical Performance Battery (/12), (mean ± SD)	7.2 ± 2.9
4 m gait speed (m/s), (mean ± SD)	0.7 ± 0.3
Severe sarcopenia (n, %)	26 (81.3%)
Malnutrition according to GLIM criteria (n, %)	17 (63%)
Charlson comorbidity index, (mean ± SD)	5 ± 2
Barthel index (/100), (mean ± SD)	91 ± 11
Lawton index (/8), (mean ± SD)	6 ± 2
Quality of Life—SarQoL (/100), (mean ± SD)	
SarQoL D1: Physical and mental health	63.9 ± 17.4
SarQoL D2: Locomotion	56.4 ± 21.0
SarQoL D3: Body composition	65.0 ± 20.0
SarQoL D4: Functionality	62.8 ± 19.4
SarQoL D5: Activities of daily living	48.1 ± 22.8
SarQoL D6: Leisure activities	31.2 ± 21.4
SarQoL D7: Fears	71.9 ± 24.2
Overall QoL score	57.1 ± 17.1

Abbreviatures: GLIM: Global Leadership Initiative on Malnutrition; SarQoL: Sarcopenia Quality of Life.

**Table 2:** Key findings from the study on the effects of Ca-HMB supplementation combined with a 12-week resistance training program in older adults with sarcopenia following hospitalization due to acute illness.

Changes in Women after a 12-Week Intervention Based on an Intragroup Intention-to-Treat Analysis						
	Intervention Group (n = 14):			Control Group (n = 10):		
	Baseline	12-Week Follow-Up	*p*	Baseline	12-Week Follow-Up	*p*
Physical performance						
SPPB—Balance test (/4)	2.4 (SD 1.2)	3.3 (SD 0.8)	0.068	3.7 (SD 0.7)	3.2 (SD 0.8)	0.104
SPPB—Gait speed test (/4)	2.3 (SD 1.1)	2.9 (SD 1.2)	0.193	2.2 (SD 1.5)	2.4 (SD 1.3)	0.169
SPPB—Chair stand test (/4)	1.1 (SD 0.7)	1.8 (SD 1.0)	0.045	1.7 (SD 1.5)	1.8 (SD 1.4)	0.347
Total SPPB (/12)	5.9 (SD 2.6)	8.0 (SD 2.3)	0.025	7.6 (SD 3.3)	7.4 (SD 3.4)	0.681
4 m gait speed test (m/s)	0.6 (SD 0.3)	0.7 (SD 0.2)	0.419	0.6 (SD 0.3)	0.7 (SD 0.3)	0.485
Muscle strength						
Handgrip (kg)	12.4 (SD 4.0)	16.1 (SD 3.9)	0.042	14.8 (SD 3.8)	15.8 (SD 4.0)	0.012
Intergroup Analysis of Changes in Primary Outcomes in Women after the 12-Week Intervention						
	Intervention Group (n = 14)	Control Group (n = 10)		Mean Difference (95%CI)		*p*
Physical performance						
SPPB—Balance test (/4)	0.9 (SD 1.4)	−0.4 (0.7)		1.3 (0.3 to 2.4)		0.018
SPPB—Gait speed test (/4)	0.6 (SD 1.3)	0.2 (0.4)		0.4 (0.4 to −0.6)		0.42
SPPB—Chair stand test (/4)	0.7 (SD 0.9)	0.1 (0.3)		0.6 (−0.1 to 1.3)		0.092
Total SPPB (/12)	2.1 (SD 2.5)	−0.1 (0.8)		2.2 (0.4 to 4.0)		0.021
4 m gait speed test (m/s)	0.1 (SD 0.3)	0.3 (0.8)		−0.2 (−0.7 to 0.3)		0.409
Muscle strength						
Handgrip (kg)	3.7 (SD 5.6)	1.0 (0.9)		2.8 (−0.8 to 6.4)		0.119

Note: Intragroup analysis shown in women at 12 weeks from baseline. Mean values (and standard deviation, SD) are displayed; mean differences were evaluated with Student’s t-test for related samples. Intergroup analysis shown in women at 12 weeks from baseline. The mean change in variables at 12 weeks and at one year with respect to baseline was determined. Mean values (SD) are displayed. Significance was evaluated with Student’s *t*-test for independent samples. Abbreviations: SPPB: Short Physical Performance Battery test [26].

**Table 3:** Feasibility assessment using TELOS components.

Components ^1^	Questions to Be Considered	Expected Answer	Reported Answer	Actions to Address Potential Barriers ^1^
Technology	Is the required equipment available?	Yes	Yes	-
	Are staff properly trained to conduct the intervention?	Yes	Yes	-
Economics	Are all the costs well defined?	Yes	Yes	-
	Is the intervention expensive?	No	No	-
	Is the time cost acceptable?	Yes	Unknown	Transportation costs should be affordable for patients.
Legal requirements	Does the intervention conflict with legal requirements?	No	No	-
	Are the standards of good clinical practice followed?	Yes	Yes	-
Operational needs	Is the intervention properly defined?	Yes	Yes	-
	Do patients and relatives accept the intervention?	Yes	Unknown	Relatives must be involved in the rehabilitation process.
	Does patient enrollment represent a barrier to the accomplishment of the research objectives?	No	Yes	Educational strategies are needed to promote the importance and benefit of participating in research studies.
	Is a third party required to attend the intervention program?	No	Yes	The known benefits of exercise should be explained to the patients and their relatives.
	Are there any social limitations to the participation of older adults?	No	Yes	Additional support from professionals and relatives could be necessary.
	Are training costs assessed?	No	No	
Scheduling	Given our current experience, is the intervention realistic in a post-hospitalization period?	Yes	Unknown	The patient should be informed about the need for timely treatment of sarcopenia.
	Will the intervention result in meaningful benefits for patients?	Yes	Unknown	The benefits of HMB for the patient have been studied in a parallel research study.

^1^ Description of the technological, economic, legal, operation, and scheduling (TELOS) components; expected answers supporting the feasibility of the intervention; and actions to address potential barriers.

**Table 4:** Tolerability and safety outcomes of the intervention.

	Total Sample (n = 32)
Tolerability, n (%)	
Permanent treatment discontinuation	11 (34.4)
Treatment interruption: exercise	6 (18.8)
Treatment interruption: supplementation	4 (12.5)
Exercise dose modification	6 (18.8)
Early termination	9 (28.1)
Re-scheduling of missed sessions	6 (18.8)
Lost to follow-up	14 (43.8)
Attendance (number of attended sessions)/36, (mean ± SD)	23 ± 12.0
Nutritional compliance (number of sachets)/84, (mean ± SD)	56 ± 32.6
Safety (n, %)	
Adverse events	21 (65.6)
Adverse reactions to resistance exercise	7 (21.9)
Adverse reactions to nutritional supplementation	0
Unexpected adverse reactions	0
Serious adverse reactions	0

Note: Treatment interruption: exercise/supplementation refers to loss of three consecutive sessions/supplementation intake and includes those participants who completed at least 70% of the exercises/sachet intake. Early termination refers to situations in which the intervention was completed within the minimum requirement but was interrupted ahead of schedule.

**Table 5:** Reasons for exercise dose modification.

Type of Modification of Resistance Exercise	Explanation
Progression on the right upper limb decreased for six sessions.	Pain: Patient with osteoarthrosis of the shoulder and sequelae of right humerus fracture.
2. Progression stopped at 2 kg of weight per limb.	Weakness: Fatigue due to physical decline.
3. Progressive loading was omitted for the left shoulder abduction exercise for two weeks.	Pain associated with exercise.
4. Progressive loading was omitted for the left shoulder abduction exercise for two weeks.	Pain associated with exercise.
5. Loads were adjusted with slower progression for the left upper extremity exercises.	Weakness: upper limb lymphedema.

## Data Availability

The original contributions presented in the study are included in the article Supplementation with β-hydroxy-β-methylbutyrate after strength training in post-acute patients with sarcopenia: A randomized, double-blind placebo-controlled trial, https://doi.org/10.1016/j.archger.2023.105323 (accessed on 3 September 2015).
